# Reports of Societies

**Published:** 1876-07

**Authors:** 


### Reports of Societies.

The Twenty-sixth Annual Meeting of the Illinois State Medical Society was held in the chapel of the Industrial University, Champaign, May 16th, and continued in session three days.

FIRST DAY.

The meeting was called to order by the President, T. D. Washburn, and prayer was offered by Rev. W. G. Pierce, of Champaign, and several pieces of music were performed by the students of the University.

Dr. S. H. Birney, of Urbana, chairman of the committee of arrangements, on behalf of the committee and the citizens of Champaign and Urbana, delivered an address of welcome. Dr. J. M. Gregory, LL.D., Regent of the University, welcomed the Association to the halls, grounds and recitation rooms belonging to the University, and announced several exercises, prepared by various members of the faculty, for the entertainment of the members of the Association.

Dr. J. H. Hollister, of Chicago, replied to these addresses on behalf of the society, after which the society adjourned until afternoon.

AFTERNOON SESSION.

At the opening of the afternoon session, Dr. E. R. Willard, of Wilmington, and Dr. Worrell, of Bloomington, were appointed to serve on the Board of Censors, in place of absentees on that committee.

Dr. Massey read the report of the Committee on Practical Medicine, which elicited a very interesting debate.

Dr. Hill, of Jerseyville, read the report of the Committee on Diseases of Children, which was followed by a discussion in regard to the food for children brought up by hand, and in regard to the treatment of cholera infantum.

The report of Dr. McClelland, on Medical Jurisprudence, was read by the Secretary, Dr. T. D. Fitch.

The society adjourned until Wednesday morning.

The evening was spent in witnessing some fine experiments with the spectroscope, by Prof. Robinson; in seeing Prof. Miles show, by proper illustrative apparatus, the movements of the intercostal muscles in the human subject; and in visiting the Art Gallery, the Library, and other halls of the Industrial University.

SECOND DAY.

The Association re-assembled at 8 o'clock, and Dr. E. S. Hayes read the report on Electrical Therapeutics.

Dr. T. D. Fitch presented his resignation of the office of Permanent Secretary, which was accepted, and a vote of thanks, offered by Dr. Worrell, and passed unanimously, expressed the thanks of the society for the able and faithful manner with which he had discharged the duties of the office, so long held by him, and for the courteous manner with which he had always treated his fellow-members.

On motion of Dr. Hollister, the annual dues of members was fixed at $3.00, until otherwise ordered by the society.

Dr. F. C. Hotz read a report on Otology.

Dr. Moses Gunn presented the report on Surgery.

A supplementary report on Treatment of Diseases of the Hip-joint, was read by Dr. S. H. Birney.

AFTERNOON.

Dr. Jackson read a report on Gynecology.

Dr. Slater read an abstract of his report on Placenta Prævia.

The Nominating Committee reported in favor of Chicago as the next place of meeting, and reported a full list of officers, of which the following are the more important: For President, T. D. Fitch, Chicago; First Vice-President, S. H. Birney, Urbana; Treasurer, J. H. Hollister, Chicago; Permanent Secretary, N. S. Davis, Chicago.

The report was adopted.

Dr. Albin's report on Necrology was read, and Dr. Ransom read a volunteer paper on Suppositories.

At four o'clock the members of the Association witnessed a military drill of the students of the University, and at five a very interesting series of exercises in calisthenics by the female students of the University, under the direction of Miss Allen. In the evening the Association re-assembled in the chapel of the University to listen to the annual address of the President, and an address by the Regent of the University. Both these reports were ordered published in the Transactions of the Society, and the recommendations of the President in his address were referred to a special committee, consisting of Drs. W. W. Chambers, J. W. Freer and H. Z. Gill. The exercises of the evening were enlivened by music performed by Mr. and Mrs. Baker, and by the reading of several pieces by Miss Bryant.

THURSDAY.

Dr. Sarah Hackett Stevenson, of Chicago, read a report on the Progress of Physiology, the conclusion of which was greeted with applause.

Dr. J. H. Hollister read a volunteer paper on Chylous Urine.

Dr. D. E. Foote read a report on Obstetrics.

Dr. J. H. Hollister, the Treasurer of the Association, submitted his annual report.

The Secretary, Dr. T. D. Fitch, also submitted his annual report.

A communication from Dr. E. W. Gray, of Bloomington, giving reasons why a State Board of Health should be organized in Illinois, was read; also a communication on the same subject from the Jersey County Medical Society.

A series of resolutions, signed by Dr. E. P. Cooke, and adopted by the Woodford County Medical Society, with reference to medical education, was adopted.

Dr. Chambers moved the adoption of a resolution for the appointment of a committee to memorialize the Legislature of Illinois with reference to the passage of a law creating a State Board of Health.

After amendments suggested by Dr. Earle, and accepted by Dr. Chambers, the resolutions, as follows, were adopted:

*Resolved*, That a committee be appointed to memorialize the next Legislature on the subject of the appointment of a State Board of Health, and that, with proper modification, the act by which the Board of Health of Massachusetts was inaugurated, be submitted to the same, as a basis for the Illinois State Board.

*Resolved*, That, as members of the State Medical Society, each one shall consider himself bound to urge the propriety of a State Board of Health upon the Representatives from his district.

Dr. J. W. Freer was chosen chairman of the Memorializing Committee.

The Association proceeded to elect delegates to represent the body in the several State Associations of the Northwest, with the following result:

*Indiana*—Albin, of Neoga, and W. Brinton, of Tuscola.

*Wisconsin*—D. W. S. Caldwell, of Jo Daviess, and D. E. Foote, of Belvidere.

*Missouri*—G. W. Jones, Danville, and R. Roscolton, Peoria.

*Iowa*—S. C. Plumber, Rock Island, and T. D. Fitch, Chicago.

*Ohio*—S. H. Birney, Urbana, and O. P. Crane, Carbondale.

*Michigan*—J. H. Hollister and Edmund Andrews, Chicago.

After the adoption of a series of resolutions of thanks to the faculty of the Industrial University and the people of Champaign, the Association adjourned.

